# Rare upper gastrointestinal hemorrhage of cetuximab: A case report

**DOI:** 10.1097/MD.0000000000009391

**Published:** 2017-12-22

**Authors:** Shi-Jie Duan, Zi-Ming Gao, Peng-Liang Wang, Bao-Cheng Gong, Han-Wei Huang, Lei Luo, Xin Wang, Ya-Nan Xing, Hui-Mian Xu, Fu-Nan Liu

**Affiliations:** a Department of Surgical Oncology, The First Affiliated Hospital of China Medical University; b Department of General Surgery, Shenyang 739 Hospital, Shenyang, China.

**Keywords:** cetuximab, colorectal tumors, EGFR inhibitor, gastrointestinal hemorrhage

## Abstract

**Rationale:**

cetuximab, an epidermal growth factor receptor inhibitor, is a targeted therapeutic regimen of colorectal cancers. Several common adverse effects have been found, such as cutaneous or gastrointestinal toxicity. However, according to the articles had been published, upper gastrointestinal bleeding (UGIB) is considered to be rare and its mechanism remains unclear.

**Patient concerns:**

In this report, we presented a 42-year-old male patient with advanced recto-sigmoid cancer. After palliative operation, the patient suffered from complete upper gastrointestinal (GI) obstruction, which was induced by extensive abdominal metastasis of the tumor. Considering his poor condition, we chose the targeted drug, cetuximab, as his further treatment. But after the application of cetuximab, the UGIB immediately happened twice in this patient.

**Diagnosis:**

UGIB, as a rare complication of cetuximab, occured to the patient.

**Interventions:**

We stopped the bleeding with thrombin, hemocoagulase and somatostatin and suspended the subsequent treatment plan of cetuximab. At the same time, anti-shock treatment was given immediately.

**Outcomes:**

He was died of respiratory and circulatory failure caused by UGIB and advanced tumor eventually.

**Lessons:**

UGIB should be considered as a rare but severe complication of cetuximab. When cetuximab is applied for patients with advanced colon tumors, more cautions should be required if the patients are accompanied by upper gastrointestinal obstruction. In addition, for those patients who suffered from UGIB recently, cetuximab should be prohibited if the Rockall score ranged > 5 points.

## 1 Introduction

In recent years, targeted therapy has become one of the most burgeoning techniques under the circumstance of rapidly developing treatments of tumors.^[1–4]^ Compared with conventional chemotherapy, targeted therapy agents can selectively act on the specific site of tumor cells, thus inhibiting its proliferation or progression. Therefore, targeted therapy is more beneficial and tolerable in most patients. Cetuximab is a common targeted therapeutic agents, which serves as epidermal growth factor receptor (EGFR) inhibitor and suppresses the progression of tumor growth, invasion and metastasis.^[1]^ Since 2004, a widespread application of cetuximab has obviously improved the survival of the patients with epithelial cancer. Cutaneous toxicity, gastrointestinal (GI) toxicity, and even severe anaphylaxis are common adverse effects of cetuximab.^[4–6]^ But upper gastrointestinal bleeding (UGIB) induced by cetuximab is rarely reported. In this report, we presented a recto-sigmoid carcinoma patient suffering UGIB after application of cetuximab and explored the possible mechanism of hemorrhage by reviewing some related literatures.

## 2 Case report

**Figure 1:**
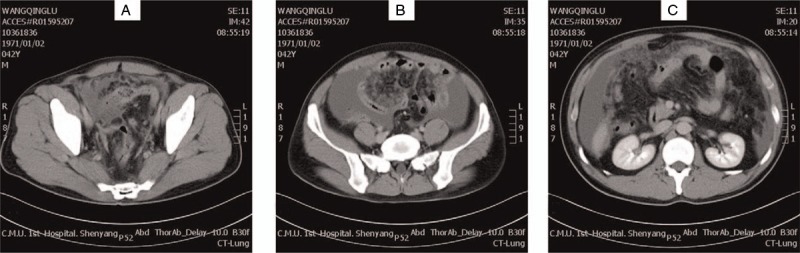
The preoperative computed tomography diagnosis of patient. (A) The intestine wall of the lesion had thickened at the recto-sigmoid junction. (B) Enhanced enteric cavity remaindered incomplete obstruction of intestine. (C) The density of intestinal mesentery and omentum increased obviously. There were amount of ascites and transferred nodes in abdominal cavity.

A 42-year-old male came to our hospital with a complaint of defecation pain for 20 days and left lower abdomen pain for 3 days. Abdominal computed tomography showed thickening intestine wall at the recto-sigmoid junction, which leaded to an obvious dilatation of proximal intestine. And the density of intestinal mesentery increased as well as omentum (Fig. 1). Through colonoscopy, we found an annular ulcer with irregular bottom and dirty surface near the recto-sigmoid junction, with pathological diagnosis of poorly differentiated adenocarcinoma. Without other obvious anomalies in the preoperative examinations, we performed an exploratory laparotomy on January 25. Massive serous ascites, a recto-sigmoid obstructive lump, and extensive nodules of implantation metastasis in abdomen, especially on the surfaces of intestinal tract and liver, were found during the operation. Considering the advanced stage of the tumor, we decided to perform a palliative Hartmann operation to relieve obstruction and adjuvant chemotherapy was chosen for following treatment. After operation, upper GI obstruction was well-relieved. But on February 10, 16 days after operation, the patient complained of discontinuous abdominal distention and pain again. Digital radiography of upper digestive tract showed a complete obstruction at the horizontal segment of duodenum, while the cavity was so narrow that the contrast agent could not pass through. We presumed that the obstruction was induced by the wide abdominal metastasis of the tumor. Conventional treatments for obstruction were performed, such as fasting, GI decompression, proton pump inhibitor, and parenteral nutrition. However, the intestinal obstruction was not relieved in the following days. Based on his situation, we assessed that the Eastern Cooperative Oncology Group Performance Status of the patient had already reached to 3 which was not suitable for high intensity of chemotherapy. So, targeted therapy was chosen as the further treatment instead of routine chemotherapy. By genetic testing, the wild type of KRAS gene had been found, for which cetuximab was very suitable. On February 27, an initial loading dose of cetuximab was given at 400 mg/m^2^; meanwhile, 250 mg/m^2^ was planned for following weeks. But on March 3, 4 days after the first course, the patient suddenly started spitting blood with the amount of 250 mL. We stopped the bleeding with thrombin, hemocoagulase, and somatostatin and suspended the subsequent treatment plan of cetuximab at the same time. On March 5, the patient threw up an 800-mL blood again while the diagnosis of UGIB had been made. Except for the previous general measures, antishock treatment was given immediately. After days of hemostasis and transfusion, the hematemesis was relieved gradually. The following endoscope revealed some old blood clot and an obvious narrowed cavity at the horizontal segment of duodenum, which mucosa was congestive and erosive seriously. Owing to the narrow cavity, endoscope could not pass through the horizontal segment of duodenum, leading to the failure in obtaining a clear image of overt bleeding. On March 14, when the condition was stable, we performed cetuximab treatment again. But a more severe hematemesis occurred immediately in just 40 h. And the patient was died of respiratory and circulatory failure caused by UGIB and advanced tumor on March 16.

We collected the blood coagulation spectrum and analyzed. However, no significant coagulation disorders were found after application of cetuximab. The changes of indexes related to coagulation during the course of treatment, including prothrombin time (PT), fibrinogen level, platelet level, and the d-dimer, were shown in Table 1. The scoring system of International Society of Thrombosis and Hemostasis (ISTH) was acknowledged to be appropriate for the diagnosis of disseminated intravascular coagulation (DIC). The total score of our patient changed from 2 to 3 points before and after the 2 application of cetuximab. They were all <5 points and did not meet the diagnosis standard of DIC. In addition, Rockall scoring system was constructed to assess the mortality of UGIB and risk of re-bleeding. In the scoring system, the risk of re-bleeding as well as death increased with the score mounting. Retrospectively using the Rockall scoring system, we concluded that the patient for first massive bleeding after endoscope scored 0 points for his age (46 years), 1 points for the systolic BP (108 mm Hg) and the pulse (126/min) recorded on May 5, 3 points for extensive abdominal metastasis found in the process of surgical exploration, 2 points for malignancy of upper GI tract diagnosed by endoscope on May 6, and 2 points for blood in upper GI tract detected by endoscope on March 6. The total score of our patient was 8 points which suggested the high-risk stage (Rockall score exceeds 5 points) with the highest risk of re-bleeding and death.

**Table 1:**
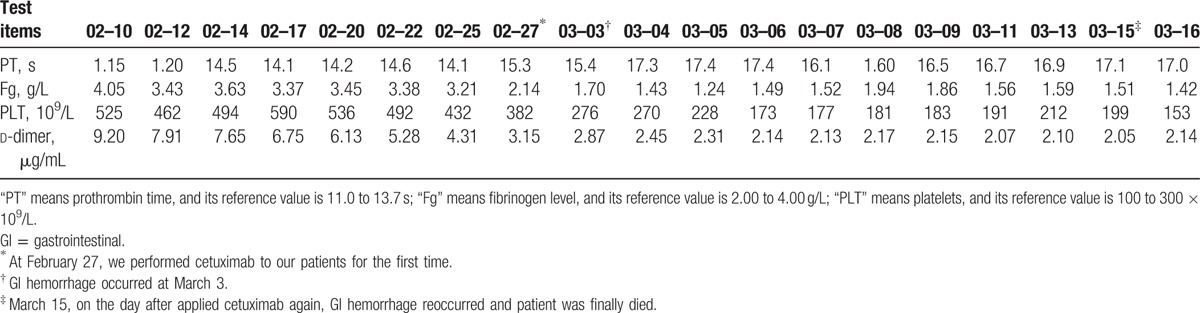
Coagulation disorder appeared after the application of cetuximab.

## 3 Discussion

As we all known, extracellular signals regulate signaling pathways through receptors on cell surface to induce proliferation or apoptosis of the cell. EGFR is such a receptor of tyrosine kinase and its overexpress has been found in GI cancers.^[1,7]^ EGFR downstream signaling pathways are important for cellular proliferation and differentiation. Cetuximab, as an EGFR inhibitor, can block the combination between EGFR and its ligands competitively, such as epidermal growth factor (EGF), resulting in inhibition of growth, invasion, and metastasis of tumor cells.

According to National Comprehensive Cancer Network (NCCN) Clinical Practice Guidelines in Oncology, cetuximab can be used as the initial treatment for the advanced or metastatic colon cancer, especially for the patients who are not suitable for high intensity of chemotherapy and have wild-type KRAS gene.^[8]^ In our report, for the reason that the intestinal-obstructed patient was diagnosed as poorly differentiated sigmoid adenocarcinoma and widespread peritoneal metastasis was suspected, we performed palliative Hartmann operation initially. Our patient's KRAS gene was proven to be wild-type and he was not suitable for high intensity chemotherapy due to his poor WHO performance status (ECOG 3 scores).^[9–11]^ Thus, we chose cetuximab as the regimen of targeted therapy for further treatment. During the course of treatment, the therapeutic schedule completely conformed to the NCCN Guidelines.

There were numerous causes of UGIB (ulcer, Mallory–Weiss tear, varices, postsurgery, etc). Considering both massive UGIB occurred in 1 month after operation, we excluded the possibility resulting from operation. Preoperative examination found no abnormalities in the upper digestive tract. Before cetuximab, a complete obstruction, resulting from wide abdominal metastasis of tumor, appeared at the horizontal segment of duodenum. Besides this obstruction, the digital radiography of upper digestive tract showed no other potential source of UGIB before the obstruction, and contrast agent as a liquid could not pass through. Above all, during the bleeding, endoscope did not revealed obvious bleeding source before the obstruction at horizontal segment of duodenum, but some old blood clot were shown at the obvious narrow section. So, we deduced that obstruction resulting from metastasis at the horizontal segment of duodenum was the source of UGIB and that ulcer, Mallory–Weiss tear, and varices were excluded. In addition, our patients recently did not receive any extra drugs which had complication of loss for GI mucosa, hemorrhage, or blood coagulation. Based on above reasons, we presumed that only metastatic neoplastic foci and cetuximab's effect are possible reasons related to the UGIB. Furthermore, there is notable detail that massive UGIB occurred twice, respectively on the 6th day and in 40th hour after application of cetuximab. It may suggest that a close temporal relationship between the application of cetuximab and UGIB should not be ignored. Therefore, although the causes of GI bleeding were not identified, we also deduced that UGIB could be a rare complication of cetuximab. Murakami et al reported 4 similar cases of advanced head and neck squamous carcinoma, which experienced gastrointestinal bleeding (GIB) within 2 weeks after the application of cetuximab–radiotherapy.^[6]^ In this article, because the radiation field did not include the GI system, it was reasonable to consider that the GIB was associated with cetuximab. However, the mechanism of GIB remains unclear. According to the related literatures and characteristics of our case, we tried to explain the GIB in 3 aspects, including reducing the protection of mucosa and vessel, shrinking lump volume of solid tumors, and continuously holding upper GI obstruction.

Cetuximab could inhibit EGFR downstream signal pathway, which is essential for mucosa.^[12,13]^ Furthermore, EGF might regulate angiogenesis both directly and indirectly. It had been reported that the EGF–EGFR signal pathway has an important effect on regulating angiogenesis especially when it comes to tumors.^[14,15]^ Several studies presented that EGFR inhibitors could suppress vascular endothelial growth factor (VEGF) expression partly. It is for the reason of angiogenesis that VEGF have a clear relationship with UGIB and could be view as a marker to assess the risk of perioperative bleeding in gastric cancer.^[16–19]^ So cetuximab could reduce the protective and renewal ability of mucosa and vessels, and increase the possibility of GI damage and hemorrhage to some extent. On the other hand, cetuximab could reduce the angiogenesis function of EGF so that tumor could not get enough supports from vessels for its growth. Therefore, cetuximab had the effect on shrinking the tumor volume. Zheng et al^[20]^ thought the application of cetuximab could shrink lump volume and form coagulative necrosis. And that could also induce GI hemorrhage, especially at the metastasis lesions. Finally, during the period of using cetuximab, our patients continuously hold upper GI obstruction leading to blood stasis and hypoxia. Blood stasis and hypoxia could induce mucosal hyperemia and increase permeability of the vessel. So, the upper GI obstruction might be another risk factor, which give rise to occurring of UGIB as a rare complication of cetuximab.

In addition, based on ISTH scoring system, the total score changed from 2 to 3 points before and after the 2 application of cetuximab. The total score was only 3 points, which was <5 points and did not meet the diagnosis standard of DIC. It should be taken into consideration that the d-dimer dropped continually after exploratory surgery and stabilized at about 2.00 to 3.00 μg/mL in our case, which contributed 2 points in the 3 points. Besides d-dimer, only the PT had the slightly significant variation in these indexes. For above reasons, the patient could not be diagnosed as coagulopathy and no tendency of DIC as we assumed. In other word, cetuximab did not induce coagulopathy and DIC.

Notably, the deadly hemorrhage appeared immediately 40 h later after the second application of cetuximab. We adopted Rockall score system to assess the risk and prognosis of the second hemorrhage. Our patient's Rockall score scored 8 points, which reached the high-risk stage (Rockall score >5 points) and suggested a high risk of re-bleeding and very poor prognosis.^[21–23]^ Therefore, for the advanced cancer patients with follow situations, including suffering from UGIB recently and Rockall score >5 points, cetuximab should be prohibited.

## 4 Conclusion

Although the mechanism is still unclear, UGIB is a rare but existing complication of cetuximab. And more cautions must be required when we use cetuximab for the patients with advanced or metastasis colon tumors and upper GI obstruction. In addition, for those patients who suffered from UGIB recently, cetuximab should be prohibited if the Rockall score exceeded 5 points.

## Acknowledgments

The authors thank the patient for his courage in the fighting with cancer. The authors also thank his family for their agreement and support in publishing the case.
